# Trends in antipsychotic and lithium use in Scandinavian countries from 2010 to 2023: a cross-country drug utilization study

**DOI:** 10.1186/s12888-026-08006-z

**Published:** 2026-03-24

**Authors:** Nina Hanieh Hakimi, Michael Due Larsen, Ivana Bojanic

**Affiliations:** 1 Faculty of Medicine and Health Sciences, Department for Clinical and Molecular Medicine, Norwegian University of Science and Technology, NTNU, Trondheim, Norway; 2 Faculty of Nursing and Health Sciences, Nord University, Levanger, Norway

**Keywords:** Antipsychotic drugs, Prevalence use, Drug utilization study

## Abstract

**Background:**

The use of antipsychotic drugs has increased in recent years, extending beyond traditional indications like schizophrenia and bipolar disorder while the use of lithium has remained stable or declined. However, current data on prescribing trends and dosages in Scandinavian countries are limited. This study aims to analyze trends in the prevalence and dosing of antipsychotics and lithium in Scandinavia from 2010 to 2023.

**Methods:**

Data were obtained from national prescription registers in Norway, Sweden, and Denmark, covering 2010–2023. For each antipsychotic class, we calculated: one-year prevalence (users per 1,000 inhabitants), therapeutic intensity (Defined Daily Doses [DDD] per 1,000 inhabitants per day), and mean dose (DDD per user per day). These metrics were analyzed overall, by sex, and by age groups.

**Results:**

In 2010–2023, overall antipsychotic use increased across all countries, with Sweden (+ 24.4%) and Norway (+ 23.7%) experiencing the largest rises, and Denmark (+ 8.1%) the smallest. Lithium use was stable in Denmark, decreased in Norway, and increased in Sweden. Use was most prevalent among women and adults aged 25–44, primarily driven by a rise in second-generation antipsychotics (SGAs), especially quetiapine. Over the period, quetiapine’s therapeutic intensity increased (e.g., Norway from 1.29 to 2.96 DDD/1,000 inhabitants/day; Sweden from 0.91 to 1.58; Denmark from 2.32 to 3.07), while its mean dose decreased (e.g., Norway from 0.63 to 0.18 DDD/user/day; Sweden from 0.35 to 0.22; Denmark from 0.34 to 0.20), indicating a trend toward low dose prescribing.

**Conclusions:**

From 2010 to 2023, prescribing patterns of antipsychotics in Scandinavia shifted toward increased use of SGAs, higher therapeutic intensity, and lower mean doses for most drugs within this class. These trends were especially notable for quetiapine and could be influenced by off-label use. These findings highlight the importance of ongoing monitoring and research that integrates prescription data with clinical information to enhance safety and promote evidence-based prescribing.

**Supplementary Information:**

The online version contains supplementary material available at 10.1186/s12888-026-08006-z.

## Background

Schizophrenia and bipolar disorder are among the top ten global contributors to disease burden and disability, affecting an estimated 64 million people worldwide [1]. Both conditions result in significant loss of life years [2, 3] and impose a substantial economic burden on society [1].Antipsychotics are a cornerstone in the management of psychosis, particularly for symptoms such as delusions and hallucinations, which are characteristic of schizophrenia [4]. In bipolar disorder, antipsychotics are primarily used to target mood stabilization [5]. This drug class is broadly divided into first-generation antipsychotics (FGAs) and second-generation antipsychotics (SGAs) (6, distinguished by their chemical structure, mechanisms of action [6] and side effect profiles [7]. Although lithium remains the most effective mood stabilizer [8] and has a well-established protective effect against suicide in bipolar disorder [8], its clinical use has plateaued in recent decades, whereas the use of antipsychotic medications has increased markedly [9, 10].

Off-label use involves prescribing medications for indications, doses, or populations not approved by regulatory agencies [11]. Recently, SGAs like quetiapine, risperidone, olanzapine, and aripiprazole have increasingly been used off-label to treat conditions such as depression and stress-related disorders [12], sleep disturbances [13], eating and personality disorders [11], dementia [14], and impulsive aggression in adolescents with ADHD [15]. However, some uses of SGAs, such as risperidone for short-term management of persistent aggression in moderate to severe Alzheimer’s dementia, have become formalized as “on-label” indications in several countries, including United Kindom, Canada, Australia and New Zealand [16]. Quetiapine has been also approved as adjunct therapy for major depressive episodes in major depression disorders with inadequate response to antidepressants in the EU and several other countries [17]. Similarly, there has been an increase in off-label use of FGAs, including haloperidol, for conditions such as acute mania [18], agitation [19], and some non-psychiatric issues like intractable hiccups [20] and chemotherapy-induced nausea and vomiting [21]. Despite their widespread use, robust long-term evidence supporting the safety and effectiveness of many off-label applications remains limited [22, 23].

Cross-sectional data from 16 countries shows a rising prevalence of antipsychotic use, especially among those aged 65 and older [24], with many prescriptions lacking documented neurological or psychiatric diagnoses. In Denmark, over one-third of prescriptions from 1997 to 2018 were issued without such diagnoses, mainly in general practice [12]. The widespread off-label use of low-dose quetiapine for non-psychotic conditions across Europe, including Scandinavia [12, 24–26], raises concerns due to often lacking medical justification and potential serious adverse effects like extrapyramidal symptoms, metabolic issues, and cardiac risks [23, 27, 28]. Recent evidence indicates a progressive shift from lithium to antipsychotics in the management of bipolar disorder, despite stable guideline recommendations favouring lithium as first-line therapy [9, 10]. Analyses encompassing eleven European countries demonstrate that lithium utilization has plateaued or declined, whereas antipsychotic prescribing has increased, with projections suggesting a continuation of this trend [9].

The three Scandinavian countries, Denmark, Norway, and Sweden, have similarly organised and comparable healthcare systems, providing tax-funded access to healthcare with relatively low charges for medication and treatment. Nevertheless, the use of antipsychotics varies markedly between these countries. During the period from 2006 to 2016, antipsychotic use increased across Scandinavia, with Norway and Denmark leading at 26.9% and 18.4%, respectively, while Sweden’s increase was only 0.9% [29]. These differences are likely to partly reflect variations in regulatory decisions, clinical guidelines and prescribing practices rather than solely true differences in the prevalence of the severe mental disorders for which these drug classes are indicated. A comparative approach across Norway, Sweden, and Denmark is warranted because these countries combine comparable healthcare organization with documented differences in psychotropic drug use, offering a unique opportunity to examine how national prescribing traditions and policy contexts shape antipsychotic and lithium utilization over time. This drug utilisation study aims to provide updated data on the prevalence and dosage patterns of antipsychotic and lithium use by genders, age groups, and countries in Scandinavia from 2010 to 2023, using publicly available national prescription registers. Descriptive drug utilization studies using standardized methodological tools, including the present study, are an essential part of the evidence base. They convert raw prescription statistics into standardized indicators that support clinical practice, guideline development, health policy, and pharmacovigilance, and help reveal potential overuse, underuse, or shifts between treatment alternatives that are not apparent from single-country prescription statistics alone.

## Method

### Study design and data sources

This drug utilization study analysed dispensed prescriptions of antipsychotics and lithium across the entire populations of Norway (approximately 5.5 million), Denmark (approximately 5.9 million), and Sweden (approximately 10.5 million) [30], spanning a 13-year period from 2010 to 2023. Data were obtained from national prescription registries in Norway [31], Sweden [32], and Denmark [33]. These registries provide publicly available data on all prescriptions dispensed by pharmacies. However, information on hospital dispensations and other inpatient care settings is not accessible.

All individuals who collected at least one or more antipsychotic and/or lithium prescriptions from a pharmacy during the study period were included. The data were anonymized and aggregated at the population level, so it was not possible to distinguish between those with single or multiple dispensations over time.

The dataset includes demographic details (age and sex), medication information (total dispensed amount in Defined Daily Doses [DDDs], number of users), and the population size for each country and year. Some variations exist between databases; for Denmark, where population data are absent from the Prescription Registry, Statistics Denmark was used [34]. Further details about data sources are available in Additional file 2, Appendix 1.

Drugs within the Anatomical Therapeutic Chemical (ATC) class N05A were included in the analysis. Lithium (N05AN01) was examined separately, as it is not considered an antipsychotic drug and is not classified as a dopamine antagonist. Antipsychotics such as acepromazine (N05AA04), prochlorperazine (N05AB04), and droperidol (N05AD08) were excluded, given their primary use as antiemetics. ATC codes with fewer than ten users or no users during 2010–2023 were also excluded from the analysis. All included antipsychotics were classified as either first-generation antipsychotics (FGAs) or second-generation antipsychotics (SGAs) based on the criteria outlined in previous studies [35–37]. Lithium was included in the analysis due to its central and guideline-supported role in the management of bipolar disorder and its frequent clinical use alongside or in comparison with antipsychotics, despite a distinct pharmacological mechanism. For details see Additional file 2, Appendix 2.

### Main measures

Antipsychotic and lithium use was evaluated using three key metrics: one-year prevalence, mean dose, and therapeutic intensity. *One-year prevalence* (users per 1,000 inhabitants) was defined as the number of individuals who filled at least one prescription during a specific calendar year. *Therapeutic intensity* was calculated as the total number of Defined Daily Doses (DDDs) used per 1,000 inhabitants per day [38]. The DDD is a standardised measure that reflects drug utilization levels rather than recommended dosing [39]. In line with WHO ATC/DDD recommendations, we used DDD per 1000 inhabitants per day, which is a standard population level indicator of therapeutic intensity that provides measure of exposure in a defined population. It provides a rough estimate of the proportion of inhabitants treated (or exposed) on an average day and allows comparisons across countries and over time. Other indicator of therapeutic intensity, DDD/patient (DDD per user) expresses average exposure among those who actually receive the drug (often referred as patients or users) and is used in individual level pharmacoepidemiological databases to approximate treatment duration (and, if actual dose equals the DDD, the number of treatment days). However, this individual‑level indicator could not be calculated in the present study due to the aggregated nature of the national prescription and sales statistics.

*Mean dose* was calculated as the total number of DDDs dispensed for each drug during a calendar year, divided by the number of users of that drug in the same year and by 365, and expressed as DDDs per user per day (total DDD pr year/365). This measure reflects the average antipsychotic exposure intensity over the year among users, rather than the prescribed daily dose on days with active treatment. To better understand prescribing patterns, especially the prevalence of low-dose use, the mean dose of quetiapine was also evaluated in milligrams per user per day across different age groups.

### Statistical analysis

The results were summarised using descriptive statistics for selected ATC groups, stratified by age categories: children (0–14 years), youth (15–24 years), young adults (25–44 years), middle-aged adults (45–64 years), older adults (65–74 years), and the elderly (75 + years). Changes over time in one-year prevalence, therapeutic intensity, and mean dose were expressed as relative percentage changes across the study period (2010–2023). Due to the absence of DDD data in Swedish prescription registers, therapeutic intensity and mean dose were manually calculated using the available information [40]. The Mann-Kendall trend test was employed to evaluate non-linear trends in drug use over time. Tau coefficients ranged from − 1 (indicating a strong decrease) to + 1 (indicating a strong increase), with p-values used to assess statistical significance at the 5% level. This non-parametric test is robust for detecting trends in time series data without assuming any specific data distribution and is resistant to outliers.

### Ethics

Only aggregate (anonymised) data at the population level from publicly available sources were used in this study, and no informed consent or ethical approval was required.

## Results

Between 2010 and 2023, the overall prevalence of antipsychotic use increased across Scandinavian countries, though with notable regional variations (see Table 1). Norway and Sweden experienced the largest relative growth across the countries, with increases of 23.7% and 24.4%, respectively. In contrast, Denmark, which already had the highest overall use in 2010, showed the smallest increase of 8.1%, compared to Norway and Sweden. By 2023, Denmark remained the leading country in overall antipsychotic prevalence, with 35.6 users per 1,000 inhabitants, followed closely by Norway with 34.9, and Sweden with 28.7 users per 1,000 inhabitants.

The use of first-generation antipsychotics (FGAs) declined significantly in all three countries. Norway saw the most substantial decrease of 65.9%, dropping from 14.7 to 5.0 users per 1,000 inhabitants. Conversely, the prevalence of second-generation antipsychotics (SGAs) increased markedly, particularly in Norway, which experienced a 143.9% rise—from 11.6 to 28.3 users per 1,000 residents. By 2023, Norway and Denmark exhibited similar SGA prevalence levels, each with approximately 28 users per 1,000 inhabitants. Lithium use remained relatively stable over time in Denmark, while it decreased in Norway (-17.1%) and increased in Sweden (+ 15.5%).

**Table 1:** Prevalence (users per 1,000 inhabitants) and relative changes (in %) in overall, first- and second-generation antipsychotics and lithium use among adults aged 15 and older in Norway, Sweden, and Denmark in 2010–2023

Antipsychotic drug Class	Country	2010	2015	2020	2023	Relative change from 2010 to 2023* (%)
		Users per 1,000 inhabitants				
First-generation						
	Norway	14.7	10.4	6.5	5.0	-65.9
	Sweden	7.6	5.9	4.5	3.7	-51.3
	Denmark	13.4	9.1	6.1	5.5	-59.2
Second-generation						
	Norway	11.6	17.4	25.1	28.3	+ 143.6
	Sweden	12.9	16.7	19.5	22.0	+ 70.4
	Denmark	17.6	22.0	26.1	28.2	+ 60.3
Total first- and second-generation antipsychotics						
	Norway	28.3	29.5	33.4	34.9	+ 23.7
	Sweden	23.0	25.3	27.0	28.7	+ 24.4
	Denmark	33.0	33.0	34.0	35.6	+ 8.1
Lithium						
	Norway	2.0	1.8	1.7	1.6	-17.1
	Sweden	2.6	2.7	3.0	3.0	+ 15.5
	Denmark	1.9	1.9	1.9	1.9	-0.6

*Kendall’s tau coefficient (τ), a non-parametric measure of association between ranked variables,

along with corresponding p-values, were included in the original analysis but have been omitted here for clarity

The analysis considered all years, although only prevalence data for three selected years are presented

All observed trends reported in the original analysis were statistically significant at the 5% level (*p* < 0.05)

### Prevalence of first- and second-generation antipsychotics and lithium by sex and age groups

**Fig. 1:**
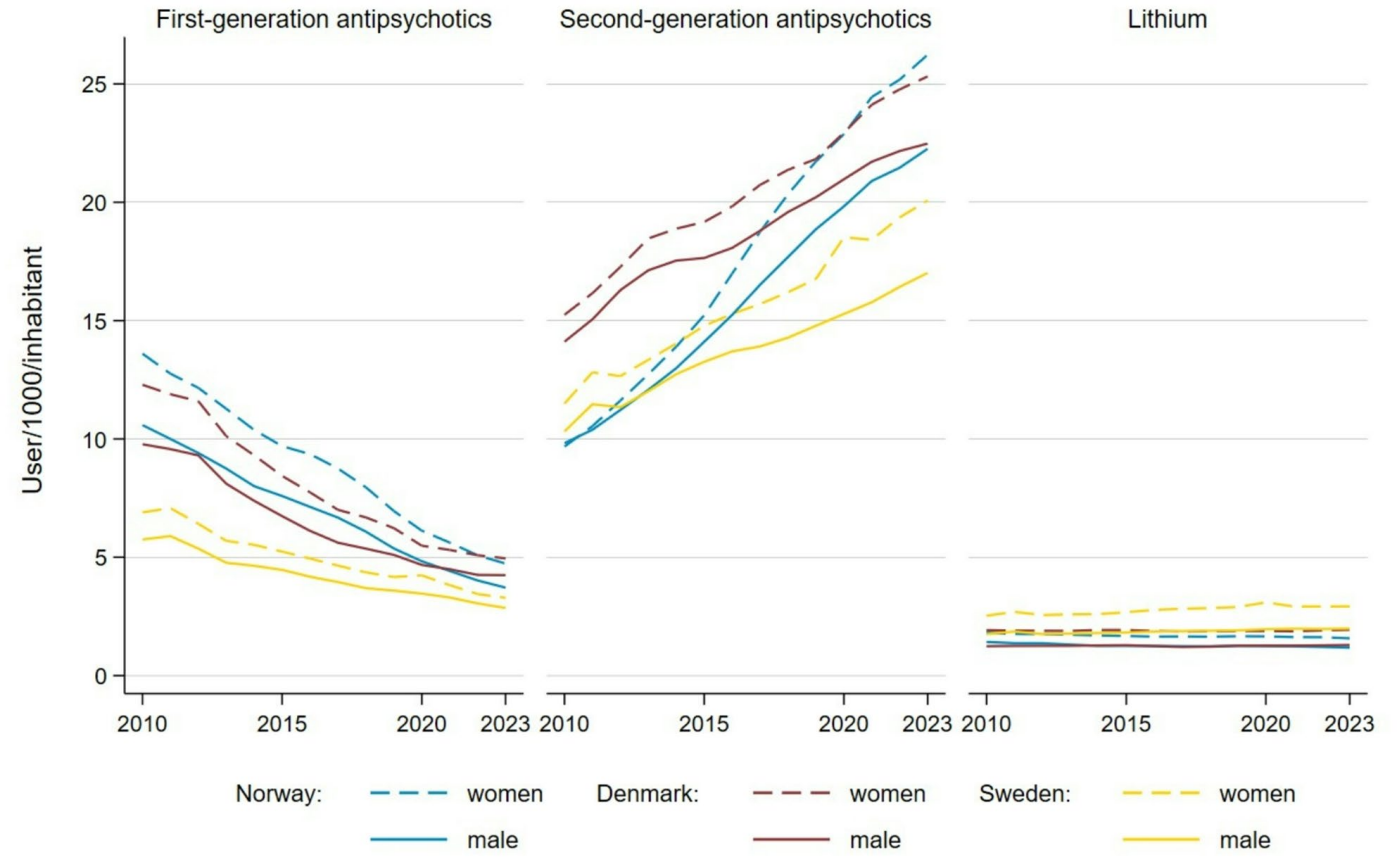
Prevalence of first-generation antipsychotics, second-generation antipsychotics, and lithium among adults aged 15 and older by sex in Norway, Sweden, and Denmark from 2010 to 2023

The use of SGAs in Norway showed a notable upward trend over time, nearly doubling among men (from 9.8 to 22.3 users per 1,000 inhabitants) and more than doubling among women (from 9.6 to 26.3 users per 1,000 inhabitants) (see Fig. 1). Initially, Norway had the lowest SGA prevalence—with values ranging from 9.8 to 26.2 in women and 9.8 to 22.3 in men between 2010 and 2014—followed by Sweden, while Denmark exhibited the highest levels. From 2015 onward, Sweden consistently exhibited the lowest SGA prevalence among the three countries for both sexes, with rates ranging from 11.5 to 20.1 in women and 10.3 to 17.0 in men, compared to Norway and Denmark.

For FGAs, the differences in prevalence between women and men were more pronounced in the early years. For example, in 2012, Norway showed prevalence rates of 13.6 in women versus 10.6 in men; Sweden had 6.9 versus 5.8; and Denmark exhibited 12.3 versus 9.8 users per 1,000 inhabitants. By 2023, these gender disparities had decreased, particularly in Norway (4.7 in women vs. 3.7 in men) and Denmark (5.0 vs. 4.2).

**Fig. 2:**
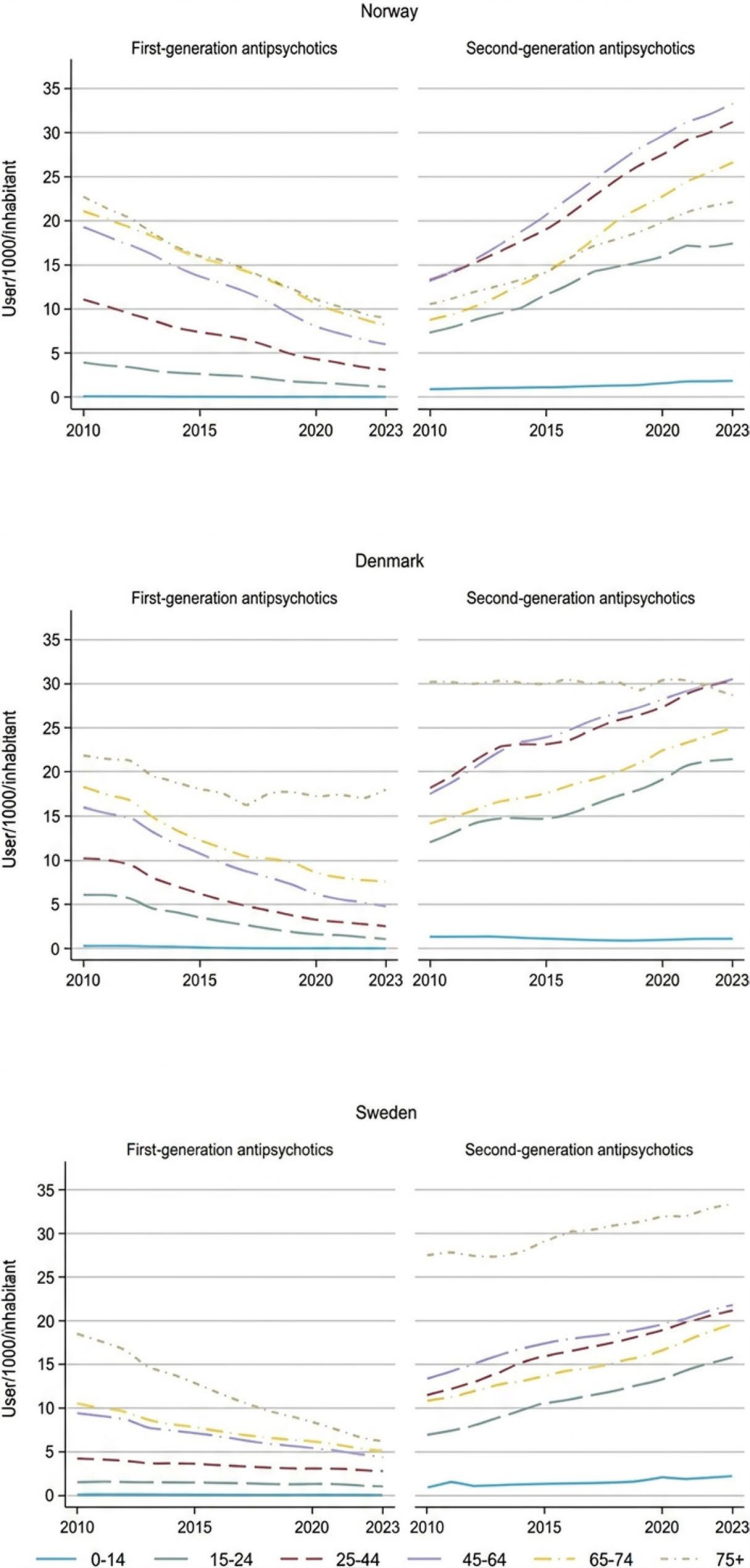
Prevalence of first- and second-generation antipsychotics in Sweden, Norway and Denmark from 2010 to 2023 by age groups

During the study period, the use of FGAs declined while the use of SGAs increased across all age groups in Norway, Sweden, and Denmark (see Fig. 2). The most significant reductions were observed among the elderly (aged 75+), with decreases of 60.6% in Norway and 66.8% in Sweden from 2010 to 2023. In Denmark, FGA use among the elderly declined steadily until 2017, reaching 16.2 users per 1,000 inhabitants, followed by a slight increase through 2019, and then stabilisation at 17.9 users per 1,000. Overall, FGA prevalence decreased with increasing age; the most substantial reductions occurred in the 45–64 (66.4%) and 65–74 (57.8%) age groups. Smaller declines were seen in the 15–24 (71.0%) and 25–44 (67.1%) groups. Notably, Norway and Denmark experienced sharper declines in FGA use among individuals aged 15–44, whereas Sweden maintained relatively stable prevalence in this younger cohort throughout the period.

Compared to Norway and Sweden, Denmark experienced a smaller relative increase in SGA use across all age groups. Among adults aged 75 and older, SGA use was highest: Sweden continued its upward trend, Denmark maintained stable levels, and Norway, despite having the lowest prevalence, showed a gradual increase. Over the study period, the most notable growth in SGA use occurred in the 15–24 age group, with a 51.6% increase, followed by the 25–44 age group, which experienced a 48.1% rise across Norway, Sweden, and Denmark. Despite differences in overall prevalence levels, the increasing trends in these age groups were remarkably similar across all three countries, albeit from different starting points.

**Fig. 3:**
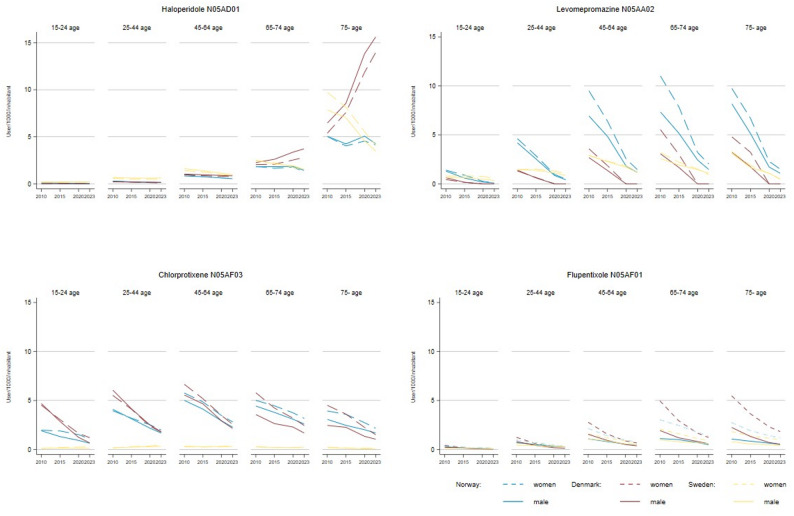
Prevalence of the four most prescribed first-generation antipsychotics in Norway, Sweden, and Denmark by age groups and sex from 2010 to 2023

From 2010 to 2023, FGA use varied across countries (Fig. 3). In Denmark, haloperidol number of users increased among elderly, from 6.4 to 15.6 per 1,000 in men and 5.3 to 14.0 in women, respectively. However, the rising annual prevalence of haloperidol among both age groups, combined with reductions in therapeutic intensity and mean dose, indicates short-term rather than permanent use (Supplementary Fig. S1), and likely results in overall reduced use. Conversely, in Sweden, it decreased from 9.0 to 3.8, and in Norway, it remained stable. Use of chlorprothixene and levomepromazine declined steadily in all three countries, with Norway showing the most significant drop in levomepromazine use among women aged 45+, from 9.8 to 1.6 per 1,000 inhabitants.

The use of SGAs remained stable overall, with quetiapine showing a consistent increase across all three countries, notably among age groups 25–44 and 45–64. Conversely, olanzapine use increased among elderly women in Sweden; however, it declined in Denmark and remained stable in Norway for both sexes. Aripiprazole use slightly increased over time, with no clear differences by sex or age groups. Risperidone use declined, especially among those aged 75 and older, across the countries.

**Fig. 4:**
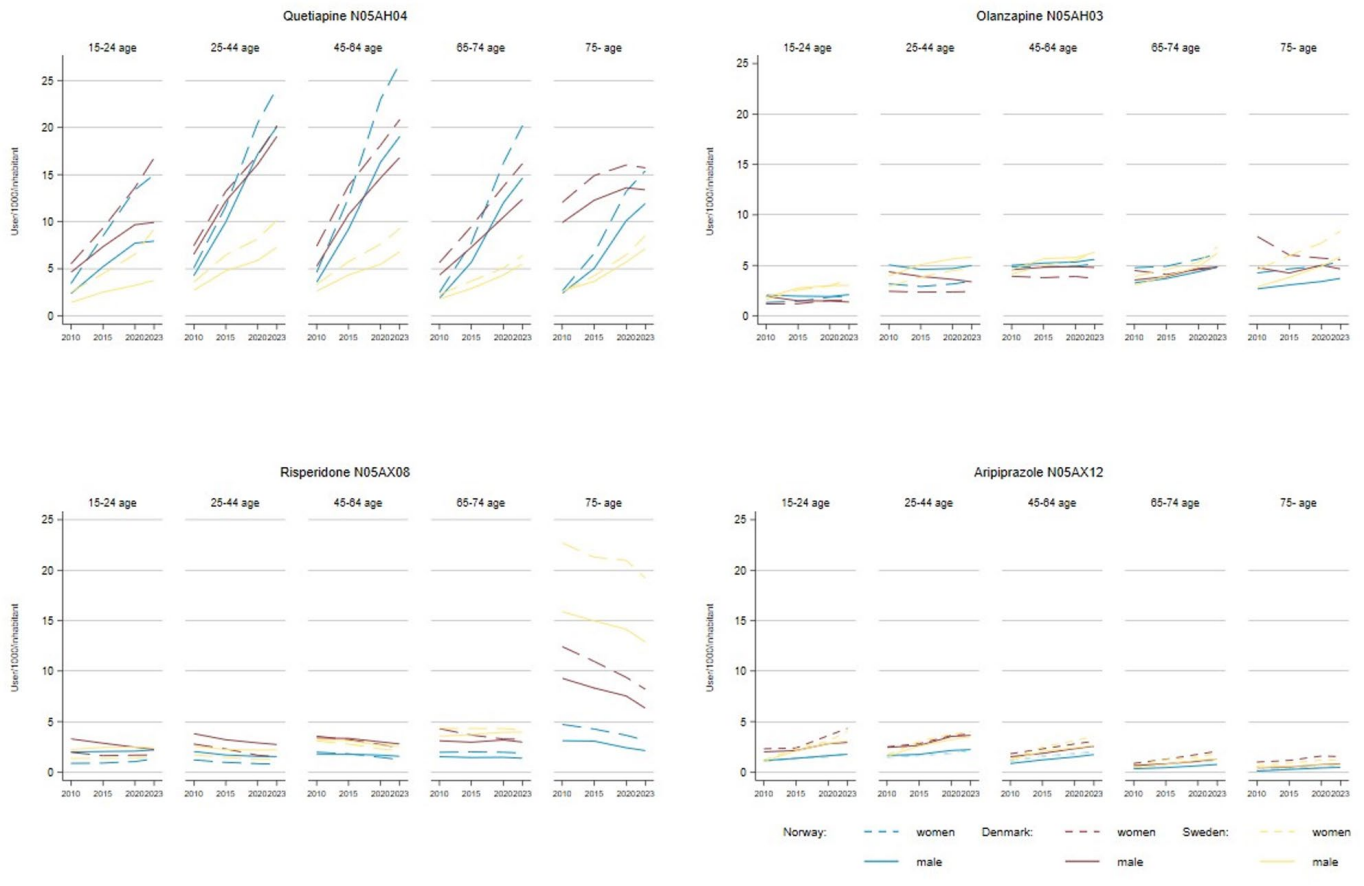
Prevalence of the four most prescribed second-generation antipsychotics in Norway, Sweden, and Denmark by age groups and sex from 2010 to 2023

Overall, use of SGA remained stable across the three countries, except for Quetiapine, which showed a consistent increase—particularly among the 25–44 and 45–64 age groups (Fig. 4). In contrast, olanzapine use increased among elderly women in Sweden but declined in Denmark and remained stable in Norway for both sexes. Aripiprazole use experienced a slight rise without significant differences by age or sex. Meanwhile, risperidone use decreased overall, most notably among individuals aged 75 and older across all three countries. For detailed information on the prevalence of FGA, SGA, and lithium use across age groups and sexes in the countries, please refer to Additional File 1, Table S1.

### Therapeutic intensity and mean doses

Therapeutic intensity for all FGAs declined across Scandinavia, with haloperidol showing significant reductions in both intensity and mean dose, most markedly in Denmark (Additional file 3; Fig. S1). Conversely, the therapeutic intensity for three out of four SGAs increased over the period, notably for quetiapine, which experienced the largest rise in Norway (+ 129.5%), followed by Sweden (+ 73.6%) and Denmark (+ 32.3%) (Additional file 3; Fig. S2). Despite this, the mean dose of quetiapine decreased sharply in Norway (− 71.4%, from 0.63 to 0.18 DDD per user per year), with similar downward trends in Denmark (− 50%) and Sweden (− 40%). Risperidone’s therapeutic intensity and mean dose gradually declined across countries, with the steepest reductions observed in Denmark (Additional file 3; Fig. S2).

**Fig. 5:**
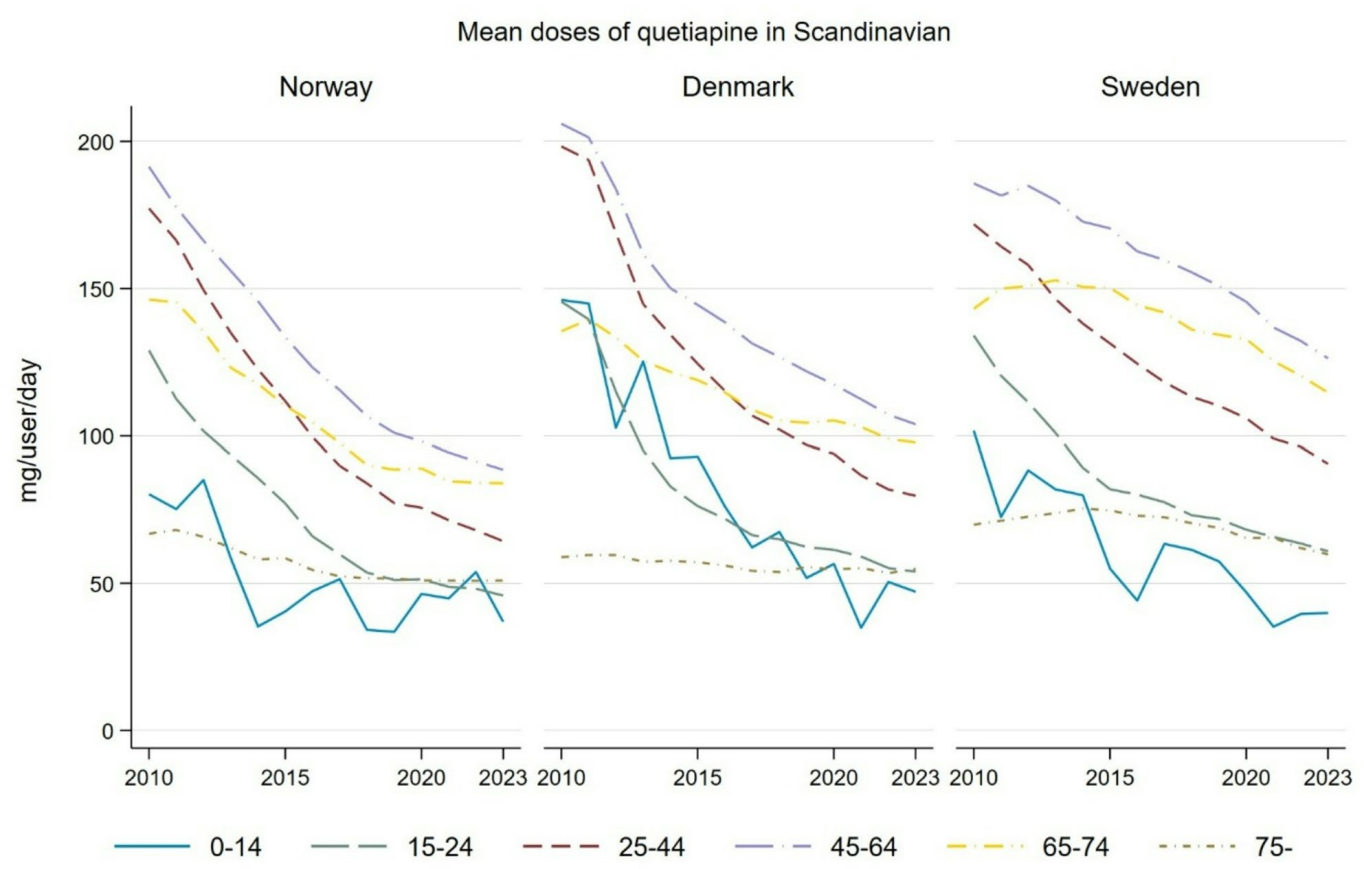
Trends in the mean doses of quetiapine (mg/user/per day), stratified by age group, in Norway, Sweden, and Denmark from 2010 to 2023

Quetiapine was the only antipsychotic to show both an increase in therapeutic intensity and a significant reduction in mean dose (Fig. 5). From 2010 to 2023, mean doses declined most among younger groups, up to 68% in children and adolescents aged 0–14 in Denmark, and less in those aged 75 and older. Norway and Denmark experienced the largest decreases in the 15–24 and 25–44 age groups (− 64.6% and − 63.7%, respectively), as well as in children and adolescents (− 68.3% in 0–14 and − 63% in 15–24). Conversely, adults aged 75 + showed only modest declines following an initial rise across all three countries.

## Discussion

This register-based drug utilisation study provides a comprehensive update on antipsychotic and lithium prescribing trends across three Scandinavian countries from 2010 to 2023. The results reveal an overall increase in antipsychotic use in all three countries, with the highest growth observed in Sweden (+ 24.4%), followed by Norway (+ 23.7%), and the smallest increase in Denmark (+ 8.1%). Throughout the study period, antipsychotic use was most prevalent among women and adults aged 25–44. The upward trend was mainly driven by a significant rise in the use of SGAs, particularly low-dose quetiapine, accompanied by a decline in FGA use. Lithium use remained relatively stable in Denmark, while it decreased in Norway and increased in Sweden.

Previous Scandinavian studies (2006–2016) showed rising antipsychotic use, especially in Norway (+ 26.9%) and Denmark (+ 18.4%), with minimal growth in Sweden (+ 0.9%) [29]. Our findings reveal a shift: Sweden, once with the smallest increase, now shows the highest growth, while Denmark’s rise has slowed. This highlights ongoing overall growth and changing country-specific patterns. Consistent with this study, a significant increase in SGA use and a decline in FGA use have been observed across Europe and beyond [41–44]. This trend is driven by various factors, including changes in prescribing practices [45], updates to clinical guidelines [46], differences in healthcare access [44], and prescribers’ preference for SGAs due to their lower risk of extrapyramidal symptoms and tardive dyskinesia compared to FGAs [47]. Our findings are consistent with evidence that lithium is increasingly being replaced by antipsychotics in the treatment of mental disorders, including bipolar disorder, despite guideline recommendations, and that it remains underused in many European countries [9, 10].

We consistently observed higher antipsychotic use among women, with the gender gap—especially for SGAs—widening in 2023 compared to 2010, notably in Norway. These findings align with broader European studies showing women are more likely than men to use psychotropic medications, regardless of sociodemographic or mental health factors [48]. Contributing factors could include women’s greater likelihood of seeking mental health care, increased sensitivity and better therapeutic response to antipsychotics, and generally lower medication doses [49]. Additionally, women have higher rates of depression, anxiety, and other psychiatric conditions compared to men, possibly due to differences in dopamine signalling [50], and are often treated with SGAs [51], which may explain their higher medication use.

Overall, from 2010 to 2023, FGA use declined across all age groups in Scandinavia, with the largest reductions among adults aged 75 and older. Prescribing trends were more stable in Denmark compared to Norway and Sweden, where quetiapine, now the most used SGA, was frequently prescribed at low doses for off-label purposes such as insomnia and dementia-related behaviours [12], despite the absence of relevant diagnoses. An exception was haloperidol, an FGA agent: despite declines in therapeutic intensity and mean dose, the number of users (per 1000 inhabitants) decreased overall. Haloperidol has increasingly been used as a benzodiazepine alternative in palliative care for Danish elderly patients with dementia [52], and with a broader decline in anxiolytic prescribing [53], there has been greater reliance on antipsychotics as sedative alternatives in older Danish population. A recent report from the Danish Health Data Authority shows that antipsychotic use in older adults with dementia has remained around 20% since 2014, that an increasing share of FGA antipsychotic prescriptions is accounted for by haloperidol used as terminal treatment, and that there is substantial regional and municipal variation in such prescribing [52]. Our aggregated prescription data do not capture diagnosis for antipsychotics and therefore cannot distinguish palliative care in dementia from other indications, but the Danish report supports the interpretation that part of the observed rise in haloperidol users in our study likely reflects short-term, end-of-life use rather than long-term antipsychotic treatment in the broader population.

Olanzapine, aripiprazole, and risperidone, all SGAs, have maintained stable prevalence, treatment intensity, and mean dosage throughout the study period. These trends likely reflect consistent adherence to established dosing guidelines [54–56] and alignment with national and international recommendations for first-line treatment of schizophrenia and severe psychotic disorders [57]. Their well-documented efficacy and favourable tolerability profiles [58] support their ongoing clinical use, demonstrating adherence to evidence-based practices in Scandinavian countries, as shown by our data. Although the Scandinavian countries have different health authorities and regulations and individual clinical guidelines, there are only minor differences between the clinical guidelines. The differences may be found in the clinical tradition and policies.

In contrast, our findings and recent studies [59, 60] reveal a substantial increase in quetiapine use across Scandinavia from 2010 to 2023, with Norway leading the rise (+ 129.5%), followed by Sweden (+ 73.6%) and Denmark (+ 32.3%). This growth is accompanied by a significant reduction in the average dose, especially among younger populations, reflecting a shift toward low-dose, off-label prescribing primarily for insomnia [59, 60]. This trend is likely driven by quetiapine’s sedative properties and increasing concerns about the risks associated with benzodiazepines and Z-hypnotics [61–63]. However, even at low doses, quetiapine carries the potential for serious adverse effects, such as metabolic disturbances and cardiovascular issues [64, 65]. Furthermore, evidence supporting its safety and efficacy for off-label uses remains limited [22, 23]. Despite these concerns, low-dose prescribing continues to rise, highlighting the urgent need for clearer guidelines and additional research to ensure safe and effective use.

### Strengths and limitations

The Scandinavian national prescription registers are robust resources for population-level analyses of medication use, characterised by high validity and minimal missing data, which enables reliable detection of long-term drug utilisation trends [66]. To our knowledge, this is the first study to provide a comprehensive, ATC/DDD-based characterization of temporal trends in both antipsychotic and lithium use across the Scandinavian region during the study period. Beyond this, the study offers an integrated overview of these trends within a single, comparable framework and yields novel, clinically interpretable indicators of treatment patterns—such as shifts between subgroups and changes in treatment intensity over time—that cannot be derived directly from the raw public statistics.

However, several important limitations must be considered. While these registers provide estimates of medication prevalence, they do not distinguish between incident (new) and continuous (ongoing) users. This conflation can obscure initiation patterns and complicate the assessment of changes in prescribing behaviour over time [67]. Additionally, the registers lack information confirming the clinical diagnosis for which a medication was prescribed, making it difficult to link drug use to specific health conditions [66]. This limitation restricts the ability to evaluate the appropriateness of use or to conduct indication-specific analyses. Another significant constraint is that prescription registers do not capture drug use in institutional settings such as hospitals or inpatient care, which may lead to an underestimation of total medication use. Long-acting injectable antipsychotics are likely underrepresented in our data, as they are administered in clinical settings and not captured in national prescription registries that record only community pharmacy dispensations.

In this study, we used the DDD to measure drug utilisation. The DDD reflects the assumed average maintenance dose per day for a drug used for its main indication in adults, but it does not necessarily correspond to the prescribed or recommended dose [39]. Consequently, actual dosing may vary depending on the clinical context—for example, antipsychotic doses differ between schizophrenia and bipolar disorder [68]. The absence of DDD data from the Swedish Prescribed Drug Register further hindered cross-country comparisons of DDD trends. Because mean dose (DDD pr year divided by 365) was averaged over the full year, values for individuals with brief treatment episodes represent low average exposure and do not reflect dosing on treatment days. Individual-level indicators of therapeutic intensity (e.g., DDD/patient) and measures of treatment duration would be informative for distinguishing long‑term versus short‑term antipsychotic use in populations with and without schizophrenia. However, such individual‑level indicators cannot be derived from the aggregated data used in this study and are therefore beyond the scope of our analyses. Since available national prescription and sales statistics do not include information on individual diagnoses, our analyses are explicitly restricted to population‑level utilization patterns rather than indication‑specific treatment of schizophrenia, bipolar disorder, or other (off‑label) indications. To address these limitations and gain more detailed insights into antipsychotic use, future research could employ observational register-based studies at the individual level, which would allow for more nuanced analyses of treatment initiation, switching, discontinuation, and the relationship between drug use and specific clinical diagnoses.

## Conclusion

In contrast to earlier studies that focused on specific groups or limited time periods, our research offers a comprehensive and current investigation of antipsychotic and lithium use in Scandinavia from 2010 to 2023, covering all ages, sexes, and drug classes. This approach delivers a more complete and up-to-date picture of prescribing patterns in the region. We found that overall antipsychotic use has generally increased in Norway, Sweden, and Denmark from 2010 to 2023. Lithium use was stable in Denmark, decreased in Norway, and increased in Sweden. While FGA prescribing declined, SGAs, especially quetiapine, increased notably, with a noteworthy shift toward lower doses, particularly among younger adults. This pattern may indicate expanding off-label use of quetiapine for non-psychotic conditions, raising concerns about safety and appropriateness. In contrast, the use of olanzapine and aripiprazole, both SGAs, remained stable and aligned with established guidelines for severe psychiatric disorders, reflecting more targeted clinical use. These evolving trends underscore the importance of ongoing vigilance and the development of clearer prescribing guidelines, particularly concerning the low-dose, off-label use of SGAs.

## Supplementary Information

Below is the link to the electronic supplementary material.

Supplementary Material 1

Supplementary Material 2

Supplementary Material 3

## Data Availability

The data used in this study are publicly available from the websites of national prescription registries. Specific statistics included in this study are available upon request from the authors.

## Abbreviations

FGAs: First-generation antipsychotics
SGAs: Second-generation antipsychotics
DDD: Daily defined dose

## References

[1] GBD 2019 Mental Disorders Collaborators. Global, regional, and national burden of 12 mental disorders in 204 countries and territories, 1990–2019: a systematic analysis for the Global Burden of Disease Study 2019. Lancet Psychiatry. 2022;9(2):137–50. 10.1016/S2215-03662100395-3. 10.1016/S2215-0366(21)00395-3 PMC8776563 35026139

[2] Hjorthøj C, Stürup AE, McGrath JJ, Nordentoft M. Years of potential life lost and life expectancy in schizophrenia: a systematic review and meta-analysis. Lancet Psychiatry. 2017;4(4):295–301. 28237639 10.1016/S2215-0366(17)30078-0

[3] Chan JKN, Tong CHY, Wong CSM, Chen EYH, Chang WC. Life expectancy and years of potential life lost in bipolar disorder: systematic review and meta-analysis. Br J Psychiatry. 2022;221(3):567–76. 35184778 10.1192/bjp.2022.19

[4] Bruijnzeel D, Suryadevara U, Tandon R. Antipsychotic treatment of schizophrenia: an update. Asian J Psychiatr. 2014;11:3–7. 25216917 10.1016/j.ajp.2014.08.002

[5] Fountoulakis KN, Vieta E, Sanchez-Moreno J, Kaprinis SG, Goikolea JM, Kaprinis GS. Treatment guidelines for bipolar disorder: a critical review. J Affect Disord. 2005;86(1):1–10. 15820265 10.1016/j.jad.2005.01.004

[6] Chokhawala K, Stevens L. Antipsychotic Medications. StatPearls. Treasure Island (FL): StatPearls Publishing; 2025. 30137788

[7] Leucht S, Cipriani A, Spineli L, Mavridis D, Orey D, Richter F, et al. Comparative efficacy and tolerability of 15 antipsychotic drugs in schizophrenia: a multiple-treatments meta-analysis. Lancet. 2013;382(9896):951–62. 23810019 10.1016/S0140-6736(13)60733-3

[8] Airainer M, Seifert R. Lithium, the gold standard drug for bipolar disorder: analysis of current clinical studies. Naunyn Schmiedebergs Arch Pharmacol. 2024;397(12):9723–43. 10.1007/s00210-024-03210-8. 38916833 10.1007/s00210-024-03210-8 PMC11582333

[9] Bindel LJ, Seifert R. Evidence of lithium underuse in bipolar disorder: analysis of lithium and antipsychotic consumption, prediction of future trends, regional disparities and indicators of rational and inappropriate use in Europe. Naunyn Schmiedebergs Arch Pharmacol. 2025;398(12):18049–70. 10.1007/s00210-025-04389-0. 40580313 10.1007/s00210-025-04389-0 PMC12678625

[10] Kessing LV, Vradi E, Andersen PK. Nationwide and population-based prescription patterns in bipolar disorder. Bipolar Disord. 2016;18(2):174–82. 10.1111/bdi.12371. 26890465 10.1111/bdi.12371

[11] Vaz RP, Martins J, Costa AL, Brás J, Sousa R, Almeida E, et al. Off-label use of atypical antipsychotics—where are we? Eur Psychiatry. 2023;66(1).

[12] Højlund M, Andersen JH, Andersen K, Correll CU, Hallas J. Use of antipsychotics in Denmark 1997–2018: a nation-wide drug utilisation study with focus on off-label use and associated diagnoses. Epidemiol Psychiatr Sci. 2021;30:e28. 33820580 10.1017/S2045796021000159 PMC8170176

[13] Thompson W, Quay TAW, Rojas-Fernandez C, Farrell B, Bjerre LM. Atypical antipsychotics for insomnia: a systematic review. Sleep Med. 2016;22:13–7. 27544830 10.1016/j.sleep.2016.04.003

[14] Nørgaard A, Jensen-Dahm C, Gasse C, Hansen HV, Waldemar G. Time trends in antipsychotic drug use in patients with dementia: a nationwide study. J Alzheimers Dis. 2016;49(1):211–20. 26444790 10.3233/JAD-150481

[15] Birnbaum ML, Saito E, Gerhard T, Winterstein A, Olfson M, Kane JM, et al. Pharmacoepidemiology of antipsychotic use in youth with ADHD: trends and clinical implications. Curr Psychiatry Rep. 2013;15(8):382. 23881713 10.1007/s11920-013-0382-3 PMC4010184

[16] Yunusa I, El Helou ML. The Use of Risperidone in Behavioral and Psychological Symptoms of Dementia: A Review of Pharmacology, Clinical Evidence, Regulatory Approvals, and Off-Label Use. Front Pharmacol. 2020;11:596. 10.3389/fphar.2020.00596. 32528275 10.3389/fphar.2020.00596 PMC7256877

[17] Maan JS, Ershadi M, Khan I et al. Quetiapine. In *StatPearls* [Internet]. StatPearls Publishing. (2023). https://www.ncbi.nlm.nih.gov/books/NBK459145/. 29083706

[18] Pacchiarotti I, Anmella G, Colomer L, Vieta E. How to treat mania. Acta Psychiatr Scand. 2020;142(3):173–92. 33460070 10.1111/acps.13209

[19] Ostinelli EG, Brooke-Powney MJ, Li X, Adams CE. Haloperidol for psychosis-induced aggression or agitation (rapid tranquillisation). Cochrane Database Syst Rev. 2017;7(7):CD009377. 28758203 10.1002/14651858.CD009377.pub3 PMC6483410

[20] Moretto EN, Wee B, Wiffen PJ, Murchison AG. Interventions for treating persistent and intractable hiccups in adults. Cochrane Database Syst Rev. 2013;1:CD008768. 10.1002/14651858.CD008768.pub2 PMC6452787 23440833

[21] Gupta K, Walton R, Kataria SP. Chemotherapy-induced nausea and vomiting: pathogenesis, recommendations, and new trends. Cancer Treat Res Commun. 2021;1(26):100278. 10.1016/j.ctarc.2020.100278 33360668

[22] Haw C, Stubbs J. Off-label use of antipsychotics: are we mad? Expert Opin Drug Saf. 2007;6(5):533–45. 17877441 10.1517/14740338.6.5.533

[23] McKean A, Monasterio E. Off-label use of atypical antipsychotics: cause for concern? CNS Drugs. 2012;26(5):383–90. 22448598 10.2165/11632030-000000000-00000

[24] Hálfdánarson Ó, Zoëga H, Aagaard L, Bernardo M, Brandt L, Fusté AC, et al. International trends in antipsychotic use: a study in 16 countries, 2005–2014. Eur Neuropsychopharmacol. 2017;27(10):1064–76. 28755801 10.1016/j.euroneuro.2017.07.001

[25] Kamphuis J, Taxis K, Schuiling-Veninga CC, Bruggeman R, Lancel M. Off-label prescriptions of low-dose quetiapine and mirtazapine for insomnia in the Netherlands. J Clin Psychopharmacol. 2015;35(4):468–70. 26035053 10.1097/JCP.0000000000000338

[26] Peters EM, Bowen R, Balbuena L. Low-dose quetiapine for major depressive disorder and sleep improvement. J Clin Psychopharmacol. 2020;40(5):500–2. 32796393 10.1097/JCP.0000000000001262

[27] Neubert A, Wong IC, Bonifazi A, Catapano M, Felisi M, Baiardi P, et al. Defining off-label and unlicensed use of medicines for children: results of a Delphi survey. Pharmacol Res. 2008;58(5–6):316–22. 18852048 10.1016/j.phrs.2008.09.007

[28] Stroup TS, Gray N. Management of common adverse effects of antipsychotic medications. World Psychiatry. 2018;17(3):341–56. 30192094 10.1002/wps.20567 PMC6127750

[29] Højlund M, Pottegård A, Johnsen E, Kroken RA, Reutfors J, Munk-Jørgensen P, et al. Trends in utilization and dosing of antipsychotic drugs in Scandinavia: comparison of 2006 and 2016. Br J Clin Pharmacol. 2019;85(7):1598–606. 30927284 10.1111/bcp.13945 PMC6595354

[30] Dyvik EH. Population in the Nordic countries from 2000 to 2024 [Internet]. Statista; 2024 [cited 2025 Jan 12]. Available from: https://www.statista.com/statistics/1296240/nordics-total-population/.

[31] Furu K. Establishment of the nationwide Norwegian Prescription Database (NorPD)—new opportunities for research in pharmacoepidemiology in Norway. Norsk Epidemiol. 2008;18(2).

[32] Wettermark B, Hammar N, Fored CM, Leimanis A, Otterblad Olausson P, Bergman U, et al. The new Swedish Prescribed Drug Register—opportunities for pharmacoepidemiological research and experience from the first six months. Pharmacoepidemiol Drug Saf. 2007;16(7):726–35. 16897791 10.1002/pds.1294

[33] Rosenkrantz O, Wheler J, Westphal Thrane MC, Pedersen L, Sørensen HT. The Danish National Hospital Medication Register: a resource for pharmacoepidemiology. Clin Epidemiol. 2024;16:783–92. 39559743 10.2147/CLEP.S487838 PMC11572433

[34] Statistics Denmark. Population—the mobile statbank [Internet]. Copenhagen: Statistics Denmark; [cited 2025 Jan 2]. Available from: https://www.statistikbanken.dk/20021.

[35] Fink-Jensen A, Nielsen J. Klassifikation af antipsykotika [Internet]. Pro Medicin DK; 2024 [cited 2025 Jan 12]. Available from: https://pro.medicin.dk/Laegemiddelgrupper/Grupper/237000#a000.

[36] Schneider-Thoma J, Chalkou K, Dörries C, Bighelli I, Ceraso A, Huhn M, et al. Comparative efficacy and tolerability of 32 oral and long-acting injectable antipsychotics for the maintenance treatment of adults with schizophrenia: a systematic review and network meta-analysis. Lancet. 2022;399(10327):824–36. 35219395 10.1016/S0140-6736(21)01997-8

[37] Norsk legemiddelhåndbok. L 5.2 Antipsykotika [Internet]. Norsk legemiddelhåndbok; 2024 [updated 2024; cited 2024 Oct 5]. Available from: https://www.legemiddelhandboka.no/L5.2/Antipsykotika.

[38] World Health Organization. Guidelines for ATC classification and DDD assignment. Oslo: Norwegian Institute of Public Health; 2024.

[39] World Health organization. defined daily dose (DDD) [Internet]. Geneva: World Health Organization. 2024 [cited 2024 Oct 12]. Available from: https://www.who.int/tools/atc-ddd-toolkit/about-ddd.

[40] Hollingworth S, Kairuz T. Measuring medicine use: applying ATC/DDD methodology to real-world data. Pharm (Basel). 2021;9(1):60. 10.3390/pharmacy9010060 PMC8006033 33802774

[41] Verdoux H, Tournier M, Bégaud B. Antipsychotic prescribing trends: a review of pharmaco-epidemiological studies. Acta Psychiatr Scand. 2010;121(1):4–10. 20059452 10.1111/j.1600-0447.2009.01425.x

[42] Montastruc F, Bénard-Laribière A, Noize P, Pambrun E, Diaz-Bazin F, Tournier M, et al. Antipsychotics use: 2006–2013 trends in prevalence and incidence and characterization of users. Eur J Clin Pharmacol. 2018;74(5):619–26. 29307053 10.1007/s00228-017-2406-0

[43] Yasui-Furukori N, Kawamata Y, Sasaki T, Yokoyama S, Okayasu H, Shinozaki M, et al. Prescribing trends for the same patients with schizophrenia over 20 years. Neuropsychiatr Dis Treat. 2023;19:921–8. 37089914 10.2147/NDT.S390482 PMC10120815

[44] Xiang YT, Ungvari GS, Correll CU, Chiu HFK, Shinfuku N. Trends in the access to and the use of antipsychotic medications and psychotropic co-treatments in Asian patients with schizophrenia. Epidemiol Psychiatr Sci. 2016;25(1):9–17. 26289066 10.1017/S2045796015000694 PMC6998674

[45] Leucht S, Huhn M, Davis JM. Should ‘typical’, first-generation antipsychotics no longer be generally used in the treatment of schizophrenia? Eur Arch Psychiatry Clin Neurosci. 2021;271(8):1411–3. 34586466 10.1007/s00406-021-01335-y PMC8563551

[46] Correll CU, Martin A, Patel C, Benson C, Goulding R, Kern-Sliwa J, et al. Systematic literature review of schizophrenia clinical practice guidelines on acute and maintenance management with antipsychotics. Schizophr Res. 2022;8(1):5. 10.1038/s41537-021-00192-x PMC8873492 35210430

[47] Carbon M, Kane JM, Leucht S, Correll CU. Tardive dyskinesia risk with first- and second-generation antipsychotics in comparative randomized controlled trials: a meta-analysis. World Psychiatry. 2018;17(3):330–40. 30192088 10.1002/wps.20579 PMC6127753

[48] Boyd A, Van de Velde S, Pivette M, ten Have M, Florescu S, O’Neill S, et al. Gender differences in psychotropic use across Europe: results from a large cross-sectional, population-based study. Eur Psychiatry. 2015;30(6):778–88. 26052073 10.1016/j.eurpsy.2015.05.001

[49] Storosum BWC, Mattila T, Wohlfarth TD, Gispen-de Wied CC, Roes KCB, den Brink WV, et al. Gender differences in the response to antipsychotic medication in patients with schizophrenia: an individual patient data meta-analysis of placebo-controlled studies. Psychiatry Res. 2023;320:114997. 36603382 10.1016/j.psychres.2022.114997

[50] Williams OF, Coppolino M, George SR, Perreault ML. Sex differences in dopamine receptors and relevance to neuropsychiatric disorders. Brain Sci. 2021;11(9):1199. 34573220 10.3390/brainsci11091199 PMC8469878

[51] LaLonde CD, Van Lieshout RJ. Treating generalized anxiety disorder with second generation antipsychotics: a systematic review and meta-analysis. J Clin Psychopharmacol. 2011;31(3):326–33. 21508847 10.1097/JCP.0b013e31821b2b3f

[52] The danish health data authority. New users of antipsychotics in elderly with dementia [Nye brugere af antipsykotika hos ældre borgere med demens] 2025 May 5 [cited 2026 Feb. 5]: Available from: https://sundhedsdatastyrelsen.dk/data-og-registre/publikationer/laegemidler/antipsykotik.

[53] Bojanić I. Use of antidepressant and anxiolytic drugs in Scandinavian countries between 2006 and 2021: a prescription database study. Depress Anxiety. 2024;2024:5448587. 40226718 10.1155/2024/5448587 PMC11919044

[54] Drugs.com. Olanzapine dosage 2025 [Internet]. [cited 2025 May 1]. Available from: https://www.drugs.com/dosage/olanzapine.html#Usual_Adult_Dose_for_Schizophrenia.

[55] Drugs.com. Risperidone dosage 2023 [Internet]. [cited 2025 May 1]. Available from: https://www.drugs.com/dosage/risperidone.html.

[56] Drugs.com. Aripiprazole dosage 2023 [Internet]. [cited 2025 May 1]. Available from: https://www.drugs.com/dosage/aripiprazole.html.

[57] Taipale H, Puranen A, Mittendorfer-Rutz E, Tiihonen J, Tanskanen A, Cervenka S, et al. Antipsychotic use among persons with schizophrenia in Sweden and Finland, trends and differences. Nord J Psychiatry. 2021;75(5):315–22. 33331804 10.1080/08039488.2020.1854853

[58] Remington G, Addington D, Honer W, Ismail Z, Raedler T, Teehan M. Guidelines for the pharmacotherapy of schizophrenia in adults. Can J Psychiatry. 2017;62(9):604–16. 28703015 10.1177/0706743717720448 PMC5593252

[59] Abdelgadir A, Walsh R, Walsh E, Patel S. Quetiapine: off-label prescribing in a community mental health team. BJPsych Open. 2021;7(S1):S63–4.

[60] Debernard KAB, Frost J, Roland PH. Quetiapine is not a sleeping pill. Tidsskr Nor Laegeforen. 2019;139(13). 10.4045/tidsskr.19.0205 31556541

[61] Cinar BR, Ligthart SA, de Wit HA, Schellekens A, Fleuren HH, Kramers C, et al. Patterns and indications for quetiapine prescribing in Dutch primary care. BJGP Open. 2025. 10.3399/BJGPO.2024.0219 PMC12728850 40730475

[62] Atkin T, Comai S, Gobbi G. Drugs for insomnia beyond benzodiazepines: pharmacology, clinical applications, and discovery. Pharmacol Rev. 2018;70(2):197–245. 29487083 10.1124/pr.117.014381

[63] Højlund M, Rasmussen L, Olesen M, Munk-Olsen T, Pottegård A. Who prescribes quetiapine in Denmark? Br J Clin Pharmacol. 2022;88(9):4224–9. 35535441 10.1111/bcp.15388 PMC9545446

[64] Højlund M, Andersen K, Ernst MT, Correll CU, Hallas J. Use of low-dose quetiapine increases the risk of major adverse cardiovascular events: results from a nationwide active comparator-controlled cohort study. World Psychiatry. 2022;21(3):444–51. 36073694 10.1002/wps.21010 PMC9453914

[65] Alinejad SG, Williams NA, Cruess JA. Statistically significant increase in weight caused by low-dose quetiapine. Pharmacotherapy. 2010;30(10):1011–5. 20874038 10.1592/phco.30.10.1011

[66] Thygesen LC, Ersbøll AK. When the entire population is the sample: strengths and limitations in register-based epidemiology. Eur J Epidemiol. 2014;29(8):551–8. 24407880 10.1007/s10654-013-9873-0

[67] Rasmussen L, Wettermark B, Steinke D, Pottegård A. Core concepts in pharmacoepidemiology: measures of drug utilization based on individual-level drug dispensing data. Pharmacoepidemiol Drug Saf. 2022;31(10):1015–26. 35819240 10.1002/pds.5490 PMC9545237

[68] Yu CL, Carvalho AF, Thompson T, Tsai TC, Tseng PT, Hsu CW, et al. Comparison of antipsychotic dose equivalents for acute bipolar mania and schizophrenia. BMJ Ment Health. 2023;26(1). 10.1136/bmjment-2022-300546 PMC10035777 36789916

