# Supplementary material for: Trends in antipsychotic and lithium use in Scandinavian countries from 2010 to 2023: a cross-country drug utilization study
Source: BMC Psychiatry. 2026 Mar 24;26:354. doi: 10.1186/s12888-026-08006-z (PMC13126908; doi:10.1186/s12888-026-08006-z)
Supplement: Supplementary file 1 — Supplementary Material 1 [file 12888_2026_8006_MOESM1_ESM.docx]

**Appendix 1.** Description of registers, coverage and obtained data

| **Country** | **Data source** | **Coverage (period and use)** |
| --- | --- | --- |
| **Denmark** | The Danish Register of Medicinal Product Statistics, the Danish Health Data Authority (<www.medstat.dk>) | 2000-2022 (individual use and wholesale data)  Includes all prescriptions filled at community pharmacies |
| **Norway** | The Norwegian Prescription Database, the Norwegian Institute of Public Health (<www.norpd.no>) | 2004-2020 (individual use [users and amounts])  Includes all prescriptions filled at community pharmacies |
| **Sweden** | The Swedish Prescribed Drug Register, Socialstyrelsen ([www.socialstyrelsen.se](http://www.socialstyrelsen.se)) | 2006-2022 (individual use [users])  Includes all prescriptions filled at community pharmacies |
| **Sweden**  **Norway** | Nordic Medico-Statistical Committees database (NOMESCO) (<www.nhwstat.org>) | 2006-2021 (individual use [users and amounts])  Includes all prescriptions filled in the primary/hospital sector |

**Appendix 2.** Overview over first and second genertaion antipsychotics with ATC codes

| First-Generation Antipsychotics | ATC Codes* | Second-Generation Antipsychotics | ATC Codes* |
| --- | --- | --- | --- |
| Chlorpromazine | N05AA01 | Ziprasidone | N05AE04 |
| Levomepromazine | N05AA02 | Lurasidone | N05AE05 |
| Fluphenazine | N05AB02 | Clozapine | N05AH02 |
| Perphenazine | N05AB03 | Olanzapine | N05AH03 |
| Haloperidol | N05AD01 | Quetiapine | N05AH04 |
| Flupentixole | N05AF01 | Asenapine | N05AH05 |
| Chlorprothixene | N05AF03 | Amisulpride | N05AL05 |
| Zuclopenthixole | N05AF05 | Risperidone | N05AX08 |
| Pimozide | N05AG02 | Aripiprazole | N05AX12 |

ATC Codes: Anatomical Therapeutic Chemical Classification Syste
