# Supplementary material for: Trends in antipsychotic and lithium use in Scandinavian countries from 2010 to 2023: a cross-country drug utilization study
Source: BMC Psychiatry. 2026 Mar 24;26:354. doi: 10.1186/s12888-026-08006-z (PMC13126908; doi:10.1186/s12888-026-08006-z)
Supplement: Supplementary file 2 — Supplementary Material 2 [file 12888_2026_8006_MOESM2_ESM.pdf]

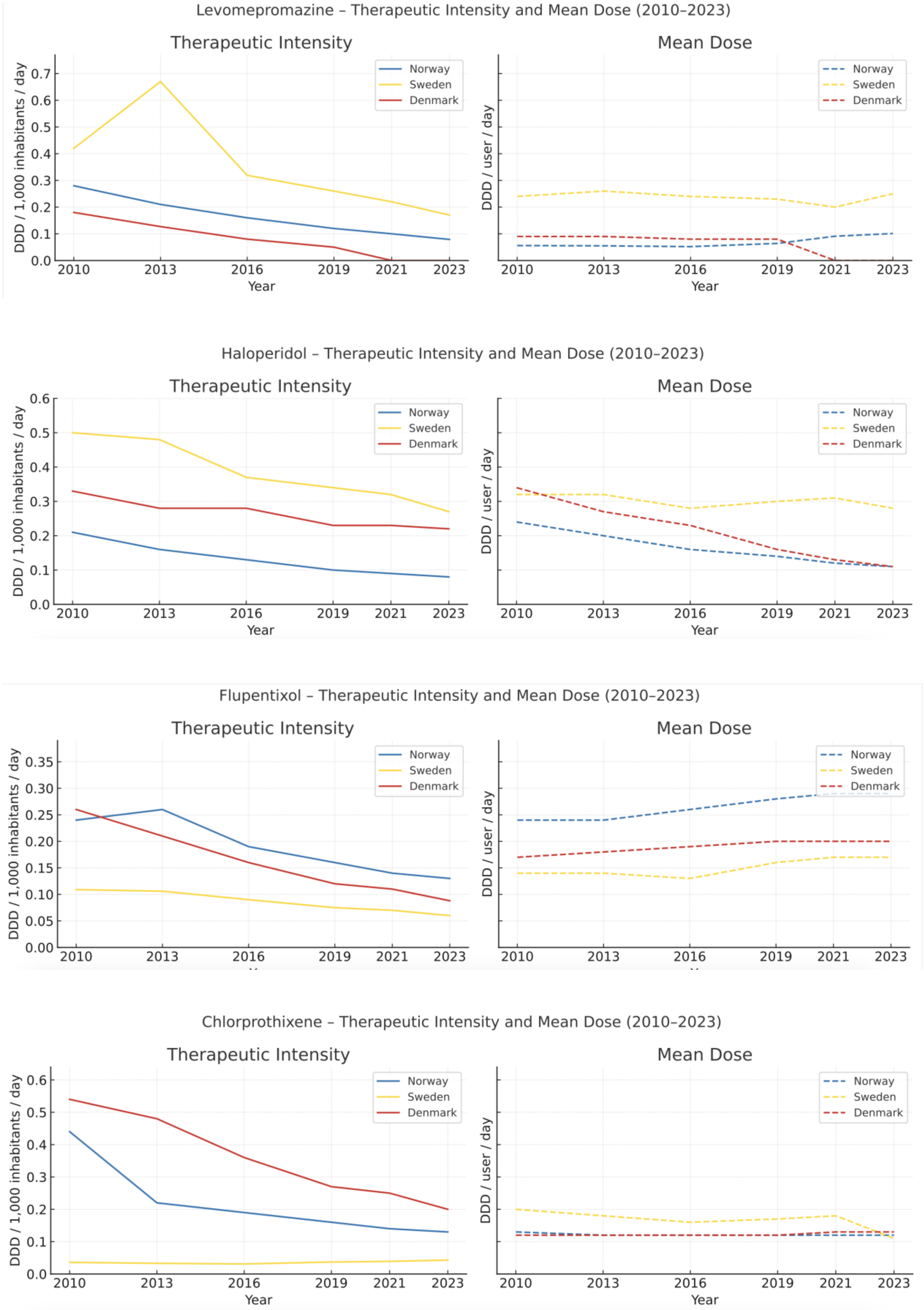

Figure S1. Trends in therapeutic intensity and mean dose of the four most prevalent first-generation antipsychotics in Norway, Sweden, and Denmark from 2010 to 2023.

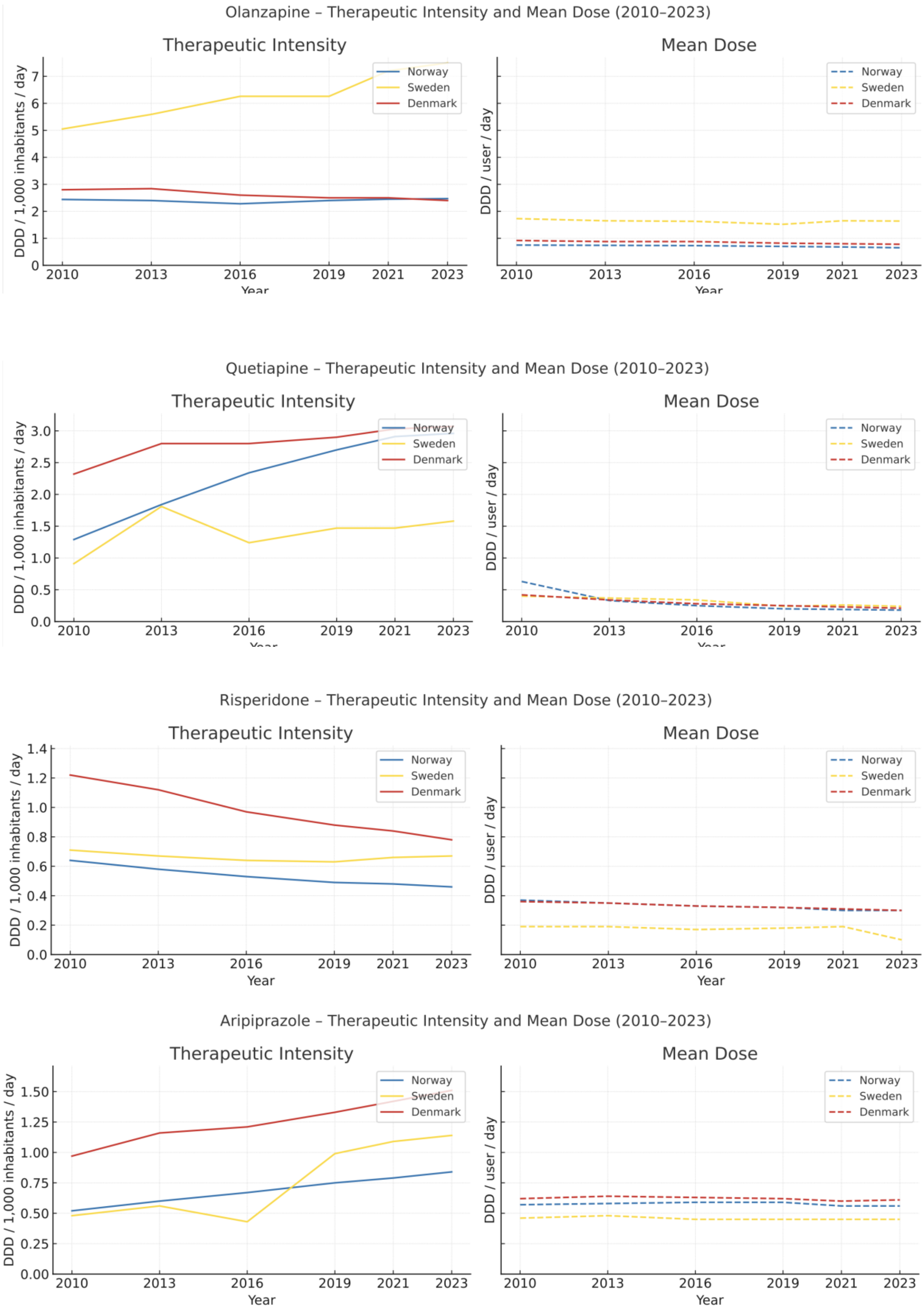

Figure S2. Trends in therapeutic intensity and mean dose of the four most prevalent second-generation antipsychotics in Norway, Sweden, and Denmark from 2010 to 2023.
