# Supplementary material for: Trends in antipsychotic and lithium use in Scandinavian countries from 2010 to 2023: a cross-country drug utilization study
Source: BMC Psychiatry. 2026 Mar 24;26:354. doi: 10.1186/s12888-026-08006-z (PMC13126908; doi:10.1186/s12888-026-08006-z)
Supplement: Supplementary file 3 — Supplementary Material 3 [file 12888_2026_8006_MOESM3_ESM.docx]

**Table S1.** Prevalence and relative changes in overall, first- and second-generation antipsychotics and lithium by age and sex in Norway, Sweden, and Denmark in 2010-2023

| Antipsychotic drug class | Country | Age | Sex | 2010 | 2015 | 2020 | 2023 | Relative change  from 2010 to 2023* (%) |
| --- | --- | --- | --- | --- | --- | --- | --- | --- |
| First-generation | Norway | 15-24 | Woman | 4.0 | 3.2 | 2.1 | 1.5 | -61.8% |
|  |  |  | Male | 3.9 | 2.2 | 1.3 | 0.9 | -77.4% |
|  |  | 25-44 | Woman | 11.3 | 7.6 | 4.6 | 3.4 | -70.0% |
|  |  |  | Male | 10.8 | 7.2 | 4.1 | 2.9 | -73.0% |
|  |  | 45-64 | Woman | 22.1 | 15.3 | 8.8 | 6.5 | -70.5% |
|  |  |  | Male | 16.8 | 12.2 | 7.2 | 5.5 | -67.4% |
|  |  | 65-74 | Woman | 24.9 | 18.4 | 12.0 | 9.2 | -63.0% |
|  |  |  | Male | 17.1 | 13.0 | 9.2 | 7.1 | -58.4% |
|  |  | 75≥ | Woman | 24.8 | 17.7 | 12.0 | 9.8 | -60.4% |
|  |  |  | Male | 19.6 | 13.6 | 10.0 | 7.9 | -59.5% |
| First-generation | Denmark | 15-24 | Woman | 5.8 | 3.6 | 1.9 | 1.4 | -75.1% |
|  |  |  | Male | 6.2 | 3.4 | 1.4 | 0.8 | -86.7% |
|  |  | 25-44 | Woman | 9.9 | 6.2 | 3.1 | 2.5 | -75.0% |
|  |  |  | Male | 10.6 | 6.2 | 3.4 | 2.6 | -75.7% |
|  |  | 45-64 | Woman | 17.6 | 11.5 | 6.4 | 5.0 | -71.8% |
|  |  |  | Male | 14.4 | 10.1 | 5.8 | 4.5 | -68.3% |
|  |  | 65-74 | Woman | 22.9 | 14.5 | 9.1 | 7.9 | -65.6% |
|  |  |  | Male | 13.4 | 9.7 | 7.9 | 7.3 | -45.5% |
|  |  | 75≥ | Woman | 25.0 | 20.4 | 17.7 | 18.2 | -27.0% |
|  |  |  | Male | 17.0 | 14.8 | 16.5 | 17.6 | 3.1% |
| First-generation | Sweden | 15-24 | Woman | 1.6 | 1.6 | 1.5 | 1.2 | -23.8% |
|  |  |  | Male | 1.4 | 1.4 | 1.1 | 0.9 | -36.5% |
|  |  | 25-44 | Woman | 4.1 | 3.6 | 3.1 | 2.8 | -30.2% |
|  |  |  | Male | 4.3 | 3.6 | 3.0 | 2.7 | -36.5% |
|  |  | 45-64 | Woman | 9.5 | 7.1 | 5.5 | 4.5 | -52.4% |
|  |  |  | Male | 9.5 | 7.1 | 5.4 | 4.2 | -55.2% |
|  |  | 65-74 | Woman | 11.8 | 8.6 | 6.4 | 5.1 | -57.0% |
|  |  |  | Male | 9.1 | 6.9 | 5.9 | 5.1 | -44.2% |
|  |  | 75≥ | Woman | 20.4 | 14.0 | 9.3 | 6.9 | -66.1% |
|  |  |  | Male | 15.7 | 11.1 | 7.1 | 5.2 | -66.8% |
| Second-generation | Norway | 15-24 | Woman | 6.9 | 12.4 | 18.2 | 20.6 | 199.2% |
|  |  |  | Male | 7.9 | 10.9 | 13.8 | 14.4 | 83.4% |
|  |  | 25-44 | Woman | 12.0 | 18.1 | 27.4 | 31.6 | 163.4% |
|  |  |  | Male | 14.6 | 19.9 | 27.6 | 30.8 | 110.4% |
|  |  | 45-64 | Woman | 13.9 | 22.4 | 32.8 | 36.8 | 164.7% |
|  |  |  | Male | 12.5 | 19.0 | 26.6 | 29.9 | 140.0% |
|  |  | 65-74 | Woman | 10.1 | 16.3 | 26.0 | 30.6 | 204.1% |
|  |  |  | Male | 7.4 | 11.9 | 19.4 | 22.5 | 206.3% |
|  |  | 75≥ | Woman | 11.9 | 16.0 | 22.4 | 25.1 | 110.7% |
|  |  |  | Male | 8.4 | 11.6 | 16.5 | 18.5 | 119.3% |
| Second-generation | Denmark | 15-24 | Woman | 11.7 | 15.1 | 21.4 | 25.9 | 121.7% |
|  |  |  | Male | 12.4 | 14.3 | 16.9 | 17.2 | 38.0% |
|  |  | 25-44 | Woman | 16.9 | 22.3 | 26.3 | 29.7 | 75.2% |
|  |  |  | Male | 19.4 | 24.0 | 28.3 | 31.0 | 60.3% |
|  |  | 45-64 | Woman | 18.6 | 25.0 | 29.4 | 31.8 | 71.3% |
|  |  |  | Male | 16.5 | 22.8 | 27.0 | 29.1 | 76.9% |
|  |  | 65-74 | Woman | 16.0 | 19.4 | 24.4 | 27.5 | 71.4% |
|  |  |  | Male | 12.2 | 15.6 | 20.3 | 22.4 | 83.7% |
|  |  | 75≥ | Woman | 33.7 | 33.1 | 32.9 | 31.2 | -7.3% |
|  |  |  | Male | 24.7 | 25.6 | 27.1 | 25.4 | 2.6% |
| Second-generation | Sweden | 15-24 | Woman | 7.0 | 11.0 | 14.4 | 18.9 | 171.3% |
|  |  |  | Male | 6.9 | 10.2 | 12.1 | 13.0 | 88.0% |
|  |  | 25-44 | Woman | 10.9 | 15.8 | 18.9 | 21.5 | 97.3% |
|  |  |  | Male | 12.0 | 16.1 | 18.9 | 21.0 | 74.7% |
|  |  | 45-64 | Woman | 13.3 | 17.6 | 20.5 | 23.1 | 73.3% |
|  |  |  | Male | 13.3 | 17.2 | 18.7 | 20.6 | 54.8% |
|  |  | 65-74 | Woman | 12.0 | 14.9 | 17.5 | 20.7 | 72.3% |
|  |  |  | Male | 9.7 | 12.5 | 15.8 | 18.6 | 91.4% |
|  |  | 75≥ | Woman | 30.9 | 33.1 | 36.6 | 38.1 | 23.2% |
|  |  |  | Male | 22.5 | 23.7 | 26.2 | 27.3 | 21.8% |
| Lithium | Norway | 15-24 | Woman | 0.4 | 0.5 | 0.6 | 0.5 | 24.6% |
|  |  |  | Male | 0.3 | 0.3 | 0.3 | 0.3 | -22.4% |
|  |  | 25-44 | Woman | 1.7 | 1.5 | 1.7 | 1.6 | -9.0% |
|  |  |  | Male | 1.5 | 1.2 | 1.3 | 1.1 | -23.8% |
|  |  | 45-64 | Woman | 3.3 | 2.9 | 2.5 | 2.3 | -28.6% |
|  |  |  | Male | 2.6 | 2.2 | 2.0 | 1.9 | -27.5% |
|  |  | 65-74 | Woman | 3.3 | 3.0 | 2.9 | 2.9 | -12.1% |
|  |  |  | Male | 2.4 | 2.2 | 2.4 | 2.1 | -13.1% |
|  |  | 75≥ | Woman | 2.0 | 2.0 | 2.0 | 1.9 | -6.1% |
|  |  |  | Male | 1.5 | 1.5 | 1.4 | 1.7 | 14.6% |
| Lithium | Denmark | 15-24 | Woman | 0.3 | 0.6 | 0.8 | 1.1 | 238.6% |
|  |  |  | Male | 0.2 | 0.3 | 0.4 | 0.5 | 185.0% |
|  |  | 25-44 | Woman | 1.5 | 2.1 | 2.2 | 2.3 | 49.3% |
|  |  |  | Male | 1.0 | 1.3 | 1.4 | 1.4 | 38.1% |
|  |  | 45-64 | Woman | 3.2 | 2.8 | 2.6 | 2.7 | -15.0% |
|  |  |  | Male | 2.3 | 2.2 | 2.0 | 2.0 | -13.9% |
|  |  | 65-74 | Woman | 3.8 | 3.2 | 2.7 | 2.6 | -30.5% |
|  |  |  | Male | 2.4 | 2.2 | 2.2 | 2.1 | -13.2% |
|  |  | 75≥ | Woman | 3.2 | 2.8 | 2.4 | 2.2 | -30.8% |
|  |  |  | Male | 1.8 | 1.6 | 1.4 | 1.4 | -20.6% |
| Lithium | Sweden | 15-24 | Woman | 0.9 | 1.2 | 1.5 | 1.5 | 69.2% |
|  |  |  | Male | 0.5 | 0.7 | 0.7 | 0.7 | 46.9% |
|  |  | 25-44 | Woman | 2.5 | 3.0 | 3.6 | 3.7 | 51.3% |
|  |  |  | Male | 1.7 | 2.0 | 2.3 | 2.4 | 43.1% |
|  |  | 45-64 | Woman | 4.1 | 4.0 | 4.2 | 4.2 | 2.1% |
|  |  |  | Male | 3.1 | 3.0 | 3.1 | 3.1 | -0.7% |
|  |  | 65-74 | Woman | 4.5 | 4.1 | 3.9 | 3.9 | -13.9% |
|  |  |  | Male | 3.1 | 2.9 | 3.0 | 3.0 | -4.1% |
|  |  | 75≥ | Woman | 3.0 | 3.1 | 3.3 | 3.2 | 9.5% |
|  |  |  | Male | 2.0 | 2.1 | 2.2 | 2.3 | 12.0% |
